# Bibliographical Notices

**Published:** 1843-04-01

**Authors:** 


### Periscope;

OR,

### CIRCUMSPECTIVE REVIEW.

"Ore trahit quodcunque potest, atque addit acervo."

### Notices of some New Works.

The Anatomy of Sleep; or, the Art of procuring Sound and Refreshing Sleep at Will. By *Edward Binns*, M.D. 8vo, pp. 394. Churchill, 1842.

This is a Cyclopædia of all the curiosities—not of literature—but of phantasies, fables, fanaticisms, deceptions, gullabilities, and Tom-fooleries, that have found record in books, and credence amongst the gobe-mouches, since the sleep of Damiens on his bed of steel, down to that of Miss Okey, under the passes of Elliotson. Of our industrious author's credulity, it is sufficient to say that he declares him a complete convert to the truth of Mesmerism!! And for what purpose was all this toil, labour, and research in collecting chaff from the four quarters of the globe, and the four winds of the sky? to *communicate* the secret of the late Mr. Gardener, for the author of these 394 pages of small type makes no pretension to the discovery!! Now this secret was communicated to hundreds of people in this metropolis, and to ourselves among others—and a more arrant piece of humbug (always excepting Mesmerism) we never beheld or heard. The poor watch-maker of Belfast (Gardener) was driven to practise this piece of charlatanism—certainly a most innocent one—by the "*res angusta domi*," rather than from any intention of deluding his patients, for the purpose of supporting existence by precarious fees. Neither was the original inventor of "Sleep at Will" able to apply the secret to himself at all times. While attending him professionally in Regent Street, we knew that he passed many a night without sleep; and as for ourselves, we have tried the panacea over and over again, without any other effect than that of invariably preventing sleep while putting the process into operation. Although we refused to give any pledge of secrecy, and assured Mr. G. that we would hold ourselves free to try the experiment on some of our patients, he nevertheless forced it on us, and in no one instance did we succeed in putting any one to sleep by the hypnotic procedure. But lest our readers should burst with impatience, we shall deliver up the grand secret at once.

"Let him turn on his right side, place his head comfortably on the pillow, so that it exactly occupies the angle a line drawn from the head to the shoulder would form and then slightly closing his lips, take rather a full inspiration, breathing as much as he possibly can through the nostrils. This, however, is not absolutely necessary, as some persons breathe always through their mouths during sleep, and rest as sound as those who do not. Having taken a full inspiration, the lungs are then to be left to their own action—that is, the respiration is neither to be accelerated nor retarded. The attention must now be fixed upon the action in which the patient is engaged. *He must depict to himself that*

*he sees the breath passing from his nostrils in a continuous stream, and the very instant that he brings his mind to conceive this apart from all other ideas, consciousness and memory depart; imagination slumbers; fancy becomes dormant; thought subdued; the sentient faculties lose their susceptibility; the vital or ganglionic system assumes the sovereignty; and, as we before remarked, he no longer wakes, but sleeps.*

"This train of phenomena is but the effort of a moment. The instant the mind is brought to the contemplation of a single sensation, that instant the sensorium abdicates the throne, and the hypnotic faculty steeps it in oblivion."

Now mark the ingenuity, or rather absurdity, of fancying the impossibility of *seeing* his own breath—and that, too, passing in a continuous stream *from* the lungs!! Yes. We can conceive that, when a person brings himself to fancy such an impossibility as this, "the sensorium abdicates its throne and the HYPNOTIC FACULTY steeps it in oblivion." Why, this gibberish equals any that is employed to convey the mummeries of Mesmerism! or the jargon of the "unknown tongues."

Near the conclusion of this most strange volume, our author tells us that he can see nothing in the human organon to prevent the possibility of "lengthening life by artificial means to *an indefinite period*," and, if so, "it must be by the subjugation of the cerebral organs to the FACULTY of sleep." True, most confiding doctor—but this will be the SLEEP OF DEATH.

After all, there is probably something in the book of a hypnotic character; for, a perusal of some twenty pages of the volume, while lounging in our armchair, after dinner, produced such a comatose sleep, with stertorous breathing, that our servant burst into the room fearing we had fallen into a fit of apoplexy. In this character we recommend the volume to our professional brethren, as preferable to morphine or even henbane. But to the ignorant its perusal will render wakefulness more wakeful by the wonderous stories that are related, and the *excerptæ* from all that has been written "*de omnibus rebus et multis aliis,*" from the sacking of Troy to the destruction of the Caisson on the Goodwin Sands.

---

ESSAYS ON DETERMINATION OF BLOOD TO THE HEAD. By *Robert Hull,* M.D. &c. &c. &c. 8vo. pp. 154. Churchill, London, 1842.

There is nearly as much of Greek and Latin in this volume as of English; nor can we always commend the style and the tone of the latter. In his address to Sir Henry Halford, he indulges in the following invective.

"Behold the reason, why, in these days of physical philosophy and natural history, the College has been malignantly opposed!

"The agitators, in the completeness of their anæsthesia, cannot appreciate any virtues, save what are to be acquired at lectures, exhibitions of mechanism, or the galleries of musea.

"They feel their inferiority to men, who are *disciplinis veteribus instructi, in libris versati, hominum eruditorum sermonibus locupletati;* and they vent splenetic obloquies."

Dr. Hull, so well instructed "disciplinis veteribus," and so great a stickler for Christian precepts, ought to have remembered that men's *motives* can only be known to themselves and their God, and that they should be judged by their *actions,* solely. Now several of these "agitators" were the most prominent of the reformers, and yet they have been gathered into the College as FELLOWS. What will these say to the anathema of Dr. Hull, who accuses them of malignancy, hatred, malice, envy, and all kinds of unworthy motives and passions!!*

What would Dr. Hull, himself say, if we followed his example in imputing *motives*, and averred that all this buttering of the College was neither more nor less than *fishing for a Fellowship.* Yet such an assertion would not be a whit more uncharitable to him than his accusations are to the reformers.

The author is a determined anti-reformer, and has almost a horror of blood-letting. We daily see the mischief of incautious phlebotomy; but we also daily witness the injury caused by neglect of early and efficient depletion. It is impossible to lay down the happy medium between the two extremes. The via tuta can only be found and trodden by observation and experience at the bed-side. The phrase "determination of blood to the head," though often used by the professional man, for the purpose of explanation to the patient, has long been exploded by physiologists. By what possible power can the heart determine or direct the stream of blood to any one organ or part, in preference to another—to the brain rather than to the liver or spleen? The supposition is perfectly absurd as well as unfounded. The abnormal fulness of blood in an organ, as the brain, is caused by the state of vessels or tissues in that part, and not on any determination to the viscus. The subjects treated of by Dr. Hull, in this little volume, are very multifarious—but not always handled in a grave, though generally erudite manner.

We can only cull out a specimen or two of Dr. Hull's manner; and it is by no means an unfavourable one.

### Teatotalism.

"That temperance will prevent the plethoric headache, every body knows. To practise is the difficulty. Is more than temperance required in drink? Is the modern system, vulgarly yclept 'Teatotalism,' the true, the salutiferous?

"The watery sect deserves great praise, statistically, nationally considered.

Αριστον μὲν ὕδωρ.

"But, in a therapeutic treatise, the question is, not how to reclaim drunkards, but how the healthy, sober man will best preserve his health? Whether by the drinking of water, or of stimulant fluid in moderation?

"The majority of medical philosophers have decided for temperance, not abstinence. The majority of, after all, a learned body—learned in *Nature.* They are prepared to forbid wine to the reckless, who know not where to stop; but for the man, mentis compoti, they think the stimulant liquids, used with caution, innocent, even useful.

"It has been asked, whether the world would not have been better without the use of stimulant drink?

"I would answer this question in morals by another, put by that great and good philosopher, whose intellect was as piercing, as his heart was devotional—whom to *read,* at the expiration of eighteen centuries, is to love with the ardor of personal friendship; whom to meet hereafter is the wish and creed of every *Christian.*

"*Terra vero fœta frugibus et vario leguminum genere, quæ cum maximâ largitate fundit, ea ferarumne, an hominum causâ gignere videtur? quid de* VITIBUS *olivetisque dicam? quarum uberimi lætissimique fructus nihil omnino ad bestias pertinent. Earum omnium rerum* HOMINUM *est usus et cura.*"—Cicero.

Throughout this essay are scattered a great number of useful hints, and judicious reflexions—not unmixed with some pedantry, dogmatism, and occasionally, even, a spice of the extravagant. Thus, speaking of insanity, the doctor observes:—

---

* Was the great reformation in the College itself a sign that opposition to it was either malignant or useless?

"The marvel is, that everybody who has *time* to think, does not run mad! In this unfathomable universe, whether viewed with the eye astronomical or microscopic, the awful so predominates, that *not to be mad* seems a special proof of the grace of God; or of a natural hebetude of soul."

To this the doctor adds, the "dogmata of the Calvinistic School," which inculcates that a *few* are selected, "for no virtues but to shew the irresistibility of their Maker," and the rest are to be damned, whether virtuous or wicked, for the same purpose of shewing the benevolence of the Deity! There is no doubt that these gloomy, not to say impious, tenets drive many weak minds mad every day. It is only astonishing that people of any intellect or reflexion could entertain such degrading ideas of the Omniscient, the Omnipotent Author of our existence and Governor of the Universe!

---

THE PRESERVATION OF THE HEALTH OF BODY AND MIND. By *Forbes Winslow*, M.R.C.S. Author of the Anatomy of Suicide, &c. 8vo. pp. 202. Renshaw, Strand, 1842.

THIS work consists of twelve Chapters—on Cold—Physiology of Death—Malaria—Longevity—Sound—Art of being Ill—Wandering of the Imagination—Diet—Asphyxia—Mental Philosophy—Mental Influence in the production of Disease—Early Indications and Treatment of Insanity. Under each of these heads Mr. Winslow contrives to introduce a great variety of miscellaneous matters, more or less directly bearing on the main object of "PRESERVING HEALTH," but interspersed with incidents and reflexions considerably beyond the horizon of the title-page. These last, however, being written in a very pleasing and even captivating style, and containing very interesting or amusing matters in themselves, will probably be read with as much avidity by the public as any other portions of the work. These, at the same time, cannot come within the limits of a review, and therefore we must select a chapter or two for notice, and for the sake of exhibiting specimens of our author's matter and manner.

The curious title of the sixth Chapter—"The Art of being Ill," arrested our attention, and we read it through most carefully. It is remarkable that the whole of it is occupied, not with the "art of being ill," but with the art of curing or alleviating disorders through the medium of the mind. It is on this account that Mr. Winslow most seriously urges medical men to make themselves well acquainted with the philosophy of the mind, or, in other words, with metaphysics. Alas! the great body of medical students have little time for metaphysical inquiries during attendance on the crowded curricula prescribed for them, and when they enter into practice (if they can get any) the "*res angusta domi*" on one hand, or the drudgery of their profession on the other, leaves them little time for metaphysical studies. But after all, where were metaphysics, or the science of mind learnt? During intercourse with mankind. And where can there be a better field than that which is presented to the medical practitioner? He daily mixes with people, some of whom are sick—some well—all agitated, more or less, by emotions of mind. He has just as good an opportunity for studying the mind as the body—and the fact is, that two of our greatest metaphysicians (Locke and Brown) were physicians. Still we are obliged to confess that our greatest medical metaphysicians were very far from being the ablest physicians. Very few, we believe, trusted their health or bodies in the hands of Locke or Brown. We apprehend that John Hunter, Astley Cooper, John Abernethy, and others that we could name, were very sorry metaphysicians. The late Dr. Armstrong considered metaphysics as little else than a jargon in which we wrangled about, of which we knew nothing. Our author, however, and several distinguished modern physicians, entertain a very high opinion of the value of mental philosophy, and, as we are very far from wishing to underrate its importance in the study and practice of physic, we recommend the work of our author to the perusal of his professional brethren.

It is to the last chapter of this volume that we shall more particularly devote our attention; but we regret to say that rarely have we met with a publication so difficult of analysis as this of Mr. Winslow. It is so pregnant with quotations and opinions from other writers, that we are bewildered at every page, as to the construction of a continuous line to travel by, or adhere to.

### The First Indications, and early Treatment of Insanity.

In this chapter our author considers it necessary to "demonstrate" the following points—

"1. The brain is the material organ of the mind.

"2. Insanity is invariably the consequence of bodily disease.

"3. On the application of the general principles of pathology to the diseases of the brain.

"4. Insanity is not a specific affection.

"5. On the importance of early treatment.

"6. Causes of insanity, insidious and apparent.

"7. On the early physical symptoms of insanity.

"8. On the early mental symptoms of insanity.

"9. Physical treatment of the incipient state.

"10. Mental treatment.

"11. On the influence of the will over the affections of the mind."

There was a time—perhaps it is not quite past—when insanity was considered a disease or disorder of the *mind* exclusively, and that physical derangement had nothing to do with the malady. This doctrine led to the most puzzling and unsatisfactory definitions of the disease.

"The natural result of this was, that medical men entirely overlooked the consideration of the early or incipient stage of insanity, for derangement was never admitted to be present until it had obtained a certain degree of development. In fact, insanity was not recognised until the affection assumed an undisguised and unmasked character. Even at the present time this mode of investigation is adopted by a few physicians who devote their attention peculiarly to this class of affections, and with the most unhappy results. It requires but little sagacity to pronounce an authoritative opinion with respect to the presence of insanity when the mental impairment is obvious and apparent to the most superficial observer; but the difficulty consists in detecting the early and delicate shades of insanity before the delusion becomes perfectly evident."

At present, it is generally admitted that the brain is the seat of the disease, as it is the organ through which MIND manifests itself. It will be still more intelligible if we look at it in this light. The manifestations of mind are the functions of the brain—and we know that all functional disorders begin insidiously, and often go on for a long time before organic changes are produced. Thus the secretion of bile is the function of the liver, and this secretion may long be in a disordered condition, before organic disease becomes manifest in the organ itself. No one would dream of thinking that bilious disorder was merely disorder of the bile itself, without having its cause in the organ which secretes that fluid.

"Having satisfactorily proved, that in every case of insanity the brain is the seat of the disease, the next question to consider is, whether the general principles of pathology which are had recourse to, with the view of elucidating the diseases of other organs, are not applicable to the brain and its disordered manifestations?

It is fallacious to suppose insanity to be a *specific* affection invariably exhibiting the same identical characteristics; like other organic diseases, it assumes many aspects, and has also an early as well as an advanced stage. The eye, the lungs, the stomach, the heart and liver, are subject to a number of affections, in all of which the peculiar function which each organ is destined to perform is obviously disordered. In every disease of the eye the vision is more or less obstructed; in every disease of the lungs the respiration is interfered with. The lungs may be inflamed, may be ulcerated, be hepatised, or effusion may take place within its substance; and in all these dissimilar disorders the breathing is manifestly deranged. How impossible would it be for us to embody within one definition all the symptoms which result from so great a variety of pulmonary diseases? The brain, like the lungs, liver and heart, is subject to a multiplicity of affections, in all of which the mental faculties are more or less affected; and yet, in investigating the phenomena of its diseases, we are influenced by principles totally at variance with those which direct us in the study and treatment of other organic maladies."

When *legally* considered, we must wait till insanity be fully developed, before we can sign a certificate or incarcerate a maniac. But *medically* contemplated, we must watch the first symptoms of mental alienation, and consider them as indicative, of incipient disorders of the brain itself, and treat them accordingly. "If the patient be neglected at this period of the attack, incurable mental derangement may result." In some institutions seven-eighths of recent cases have been cured.

But we cannot follow our author through all the divers illustrations of the symptoms, causes, nature, and treatment of incipient insanity. The work must be read, and cannot be either analyzed or epitomized. It will be a very popular work—especially among general readers—and not less popular than useful.

---

TREATISE ON THE OLEUM JECORIS ASELLI, OR COD LIVER OIL, AS A THERAPEUTIC AGENT IN CERTAIN FORMS OF GOUT, RHEUMATISM, AND SCROFULA; WITH CASES. By *John Hughes Bennett*, M.D. &c. 8vo. pp. 180. Highley, 1841.

WE cannot account for this little work having so long escaped our notice; for it is a very meritorious brochure, evincing great assiduity and research in the collection of materials scattered among a vast number of German and other Continental modern publications, respecting an article which promises to prove an important therapeutic agent in many complaints. The remedy is plentiful, easily prepared, and very moderate in price.

The cod-liver oil, though occasionally used in practice, and mentioned by British writers, has never come into that general use here which it has experienced in Germany. Drs. Barton, Percival, Darbey, Bardsley, and others, have recommended and employed it, especially in old rheumatic pains, and it appears that, some fifty years ago, fifty or sixty gallons of it were annually consumed in the Manchester Infirmary. Dr. Bardsley, in 1807, reports that "this medicine has preserved its reputation, unimpaired, in our Infirmary during the period of thirty years."

In 1822, Dr. Schenk, of Siegen, published a memoir on this subject, in Hufeland's Journal, and gives a series of very obstinate cases of rheumatism cured or relieved by the cod liver oil. Subsequently, the same author wrote a memoir in its favour for rickets. Since that time, the employment of that remedy in Germany, has been very extensive, and numerous memoirs have appeared on the subject. It has now obtained a place in the Pharmacopœias of Berlin, Hanover, Saxony, &c. &c. More recently it has been employed in France and Belgium; and, still more lately, has been noticed by Drs. Thomson, Hall, Donovan, and Christison, in this Island.

The natural and commercial history of this medicinal agent we must pass over, though minutely detailed in the second Section of the work before us. In the third Section our author takes up "the Action of the Cod Liver Oil on the Human Economy."

Carron du Villauds records the following physiological effects, as experienced in his own person, after a dose of the oil.—"A very nauseous taste not easily to be removed, somewhat resembling that of putrid fish; further, a biting sensation in the throat, in conjunction with the secretion of an adhesive saliva; which symptoms were more vehement in proportion to the impurity of the oil. The eructation of a nauseous gas continued some time after taking the dose. There followed colic pains, then light stools, together with an increased secretion of urine, both secretions possessing the characteristic smell of the oil."

Such effects, however, are not so violent in the generality of persons who take the medicine, and they vary much, according to the temperament and condition of the individual. People soon get reconciled to it, though certainly it is somewhat nauseous at first, The Laplanders, and even the Shetlanders, use it extensively as an article of diet. Dr. Bennett himself does not think it more disagreeable than castor oil, and not nearly so much so as bals. copaiba. Brefeld exhibited the medicine to upwards of a thousand people without any injurious effects. This statement, however, must be taken cum grano salis; for where there is plethora, or inflammatory diathesis, the oil is hazardous. It seldom acts much on the bowels; but it has been found to prove emmenagogue in many instances.

It is supposed to have a beneficial influence on the genital organs, when their functions are impaired; but it is in obstinate cases of gout and rheumatism that its reputation has chiefly rested, as also in scrofula.

"The MODUS OPERANDI of the oil may be said to consist in stimulating the lymphatic glands and vessels, and by these means increasing the activity of the capillary system. By its action on the former the process of assimilation is facilitated, and the appetite increased. The quality of the blood is thus improved, and so, lastly, the different organs and structures of the body become better nourished, and receive more *turgor vitalis*."

There is a considerable portion of iodine in the yellow and brown kinds of the oil, and hence, perhaps, some of their medicinal virtues.

In respect to dose, one or two tablespoonfuls of the oil may be given to an adult twice or thrice a day—the dose to be proportionably lessened according to age. Brefeld recommends it to be given with a little powdered sugar as the best corrective of the disagreeable taste. Dry biscuit answers the same purpose. A cup of coffee after the dose removes the unpleasant flavour. It should not be given in the morning fasting, as it is then more likely to disagree. About an hour after a light breakfast, is the best time, and again in the evening, after the dinner is digested. The genuine brown or yellow cod liver oil is to be used only.

### RHEUMATISM AND GOUT.

Dr. Kay, of the Manchester Infirmary, was the first in this country to employ the remedy in question in the above diseases, as the following passage in Percival's Essays will shew.

"A woman who laboured under the most excruciating rheumatism, and was an out-patient of this infirmary, being advised to rub her joints with the oil, was induced to take it at the same time internally. A few weeks restored her to the use of her limbs, and she was cured. However, little attention was paid to this case, as it was supposed that the alteration of the weather, and the medicines she had before taken, had caused the cure. About a twelvemonth afterwards, her complaints returned with double violence, and the same remedy restored her to health again. Encouraged by this second recovery, Dr. Kay, one of the physicians to the infirmary, prescribed it for other patients, in similar cases; and it answered his most sanguine expectations. Since then it has been used by the other physicians with the greatest success."

Dr. Percival himself bears testimony to the efficacy of this medicine in various maladies which had resisted other plans of treatment. Dr. Bardsley, in 1807, speaks of it as a medicine of "efficacious but limited powers."

It is curious that, notwithstanding this respectable testimony, the remedy appears to have died a natural death in this country, though it was resuscitated in Germany, by the pen of Schenk, in two memoirs, where he has extolled the oil "as a specific in gout and rheumatism."

"It heals all chronic painful affections of the human body, wherever they are seated, whether internal or external, if they have originated in rheumatism or gout, as surely and as certainly as bark cures intermittent fever, or mercury the venereal disease."

Memoirs innumerable *after* those of Schenk, were published on the Continent, —"and at present its use in these diseases is universal in Germany." There is a good deal of conflicting evidence, however, on this point, and our author makes the following remark:—

"Judging from the mass of observations which have now been published in the different German periodicals, and from what I have heard and seen connected with this subject, I am inclined to consider that the cod liver oil is more especially indicated in three distinct forms of chronic gout and rheumatism, which may be denominated the general, erratic, and local forms."

On each of these forms Dr. Bennett makes some judicious and discriminating observations, for which we must refer to the work itself. A great mass of cases of various diseases treated by the cod liver oil occupies the remainder of the volume, and these—indeed the whole of the treatise, we most strongly recommend to the attentive perusal of our professional brethren. The book is characterised by candour, discrimination, modesty and judgment.

---

THE THEORY AND PRACTICE OF MIDWIFERY. By *Fleetwood Churchill*, M.D. M.R.I.A. &c. Illustrated by upwards of 100 Wood Engravings by Bagg. London, 1842. Renshaw, pp. 479.

THIS is surely the age for treatises on Midwifery. Within the last eight or ten years we have had works on the subject by Dr. *Ramsbotham*, Dr. *Blundell*, Dr. *R. Lee*, Dr. *Rigby*, &c. all excellent in their way, and every one of which, we believe, has met with very fair encouragement from the profession. This circumstance alone shows how much more highly this branch of medicine is esteemed, and how much more zealously it is now studied, than it used to be twenty or thirty years ago. It was long a disgrace to our corporate Colleges of Physicians and Surgeons that they did not even allude to midwifery as a requisite branch of medical education on the part of the candidates for their diplomas. Fortunately a better state of things now exists, and young men are not, in the present day, sent abroad or to the provinces of our own country to practise the healing art, without perhaps having ever attended a single woman in childbed.

Of all the works recently published, none seem to us to be better suited to the wants of the student than the present one by Dr. *Churchill*. The volume is altogether admirably got up, and does great credit to the publisher as well as to the talented author. The type, although small, is most beautifully clear, and, as there are nearly 500 pages of close print, the reader can imagine what amount of information is contained within the compass of a neat duodecimo volume. It is almost unnecessary to say that the wood engravings by *Bagg* are good; most of them are capital, and will greatly diminish the student's labour in fully understanding the mechanism of parturition, whether regular or irregular.

Dr. *Churchill* has done his part remarkably well upon the whole. The only fault that we can find with his descriptions is, that he too often details the opinions—in many cases unfortunately conflicting—of different writers, instead of distinctly giving the results of his own observation and experience. In a manual upon any practical subject, the great aim of the author should be lucidly to explain what the young practitioner is to do, and how he is to form his judgement when called upon to act for himself. The collating of many authorities, especially when the practice recommended by them is very different, serves only to bewilder and mislead. We were especially struck with the truth of this in looking over the chapter on puerperal fever. It is far too elaborate in a literary point of view, and looks like a compilation rather than a didactic essay. In the chapters also on instrumental labour, we missed in many passages a certain tone of decisiveness, so to speak, in the practical instructions, that constitutes one of the great attractions of *Blundell's* writings. For example, in his description of, and remarks on the long forceps, it is not easy to discover whether Dr. C. approves of its use or not. It is an instrument that is now very little used; and certainly the less the better. If the detention of the head at the inlet arises from a mechanical impediment, no good to the child, and certainly much evil to the mother, will be caused by any attempts to bring it down by artificial means; and, on the other hand, if it arise from merely deficient expulsive energy of the uterus, there are many other simpler means which the prudent accoucheur will have recourse to, and which will very generally prove successful.

With the exception of a few such blemishes as these, the work under notice deserves very high praise; it cannot fail to be acceptable to the student, and may often be consulted by the practitioner with great advantage. We select one or two specimens, to show the style of the author and his mode of treating his subjects.

*Ordinary Duration of Pregnancy.*

The usual term of gestation in women is about nine calendar months and one week, or forty weeks, or 280 days, allowing a slight variation either way. This period, it is well known, may often be considerably shortened, and this too without great detriment to the child: but does the converse ever hold true? is the duration of pregnancy in the human subject ever considerably prolonged? There has been the greatest difference of opinion on this subject among the leading accoucheurs of this country in the present day: "Drs. Gooch, R. Blegborough, Davis, Sir C. M. Clarke, and Mr. Pennington, discrediting protracted gestation, and Drs. Granville, Conquest, Blundell, Merriman, Power, Hopkins, &c. advocating its possibility.

"Dr. Dewees remarks, 'I have had every evidence, on this side of absolute proof, that it has been prolonged to ten calendar months, as an habitual arrangement, in at least four females; that is, each went one month longer than the calculations made, from an allowance of ten or twelve days after the cessation of the last menstrual period: and from the quickening, which was fixed at four months.' Professor Desormeaux relates a case of a lady whose pregnancy lasted nine months and a fortnight. The late Professor Hamilton, of Edinburgh, declares his 'solemn conviction, that he has met with at least twelve cases, in the course of practice, where there could not be the shadow of doubt of the protraction of human pregnancy beyond the ordinary period.' M. Velpeau has recorded nine cases of the kind.

"To these authorities may be added the names of Hervey, Smellie, Zacchias, La Motte, Le Roi, Le Bas, Foderè, Capuron, Gardien, Murat, &c.

"Dr. Montgomery relates two cases in his work, one of which came under my observation; in the first, the gestation continued two hundred and ninety-one days, and in the second, forty-one weeks and two or three days at least. I have referred to some of the cases on record, because, the question being chiefly one of authority, positive evidence must infinitely outweigh mere negation.

"An additional argument has been deduced from the irregularity of the period of gestation among cattle. According to the researches of M. Tessier: out of 160 cows, 14 calved from 8 months to 8 months and 26 days; 3 at 270 days; 50 from 270 to 280 days; 68 from 280 to 290 days; 20 at 300, and 5 at 308 days; the extremes being thus 67 days apart. Of 102 mares, 3 foaled on the 311th day; 1 on the 314th: 1 on the 325th; 1 on the 326th; 1 on the 330th; 47 from 340 to 350 days; 25 from 350 to 360; 21 from 360 to 377, and 1 on the 394th day; the extremes being 83 days. With sows, the extremes were 15 days: and with rabbits (out of 139 cases) 7 days."

Dr. *Churchill* himself leans to the latter of the two opinions: viz. that gestation may be protracted for some time beyond the ordinary period, and quotes the very sensible remark of his friend Dr. *Montgomery* that, we "cannot imagine why gestation should be the only process, connected with reproduction, for which a total exemption from any variation in its period should be claimed."

*Induction of Premature Labour.*

The following summary of the various methods of performing this necessary, though highly responsible, operation, will be read with interest.

"1. Abdominal frictions, and manipulation, with warm baths, &c. have been advised, but they very rarely succeed, their supposed advantage being the absence of unnecessary irritation.

"2. Separating the membranes for two or three inches around the os uteri, will frequently bring on labor; and as this is the closest imitation of natural labour, it has been preferred by many. Dr. Hamilton remarks, 'that he is now convinced, from the experience of the last ten years, that if there be a sufficient portion of the decidua separated from the cervix uteri, there is no occasion for the introduction of the open male catheter,' *i. e.* for puncturing the membranes. Dr. Conquest considers it as effectual as the other methods, and much safer for the infant, as saving it from pressure during the pains. If it fail, we can still have recourse to the third plan.

"3. The membranes may be ruptured, either directly or obliquely. For this purpose Wenzel, Ritgen, Klugè, and others, have invented appropriate instruments; but a female catheter may be used, or a piece of wire, or a canula having concealed within it a spring trocar. Care must be taken to wound neither the mother nor child.

"This plan was adopted in Mampe's and Spoendli's cases; in 36 of Dr. F. H. Ramsbotham's—(of these 21 children were born alive, and 19 ultimately lived); and from its greater certainty, it has been preferred by most practitioners.

"4. MM. Brünninghausen and Klugè have proposed and practised, with great success, the dilatation of the os uteri, by means of a piece of sponge placed within it, and maintained there by a plug in the vagina."

(*Velpeau* gives a decided preference to the use of the plug: he says that the irritation so produced is not only more certain, but is also more permanent, progressive and regular, than by the use of any of the other methods.)

"5. Ergot of rye is now pretty generally supposed to have the power of originating uterine contraction, and if this be the case, it will probably be found to be the most effectual and safe mode of inducing premature labor, because we can preserve to the child the safeguard of the liquor amnii, which is of the greatest importance.

"Dr. F. H. Ramsbotham has mentioned many cases in which it was tried for this purpose. Labor was brought on by its use alone, at the seventh or eighth month, in twenty-six cases, without interfering with the membranes of the os uteri. All the mothers recovered, and 12 of the children were born alive, and 14 still-born. Of the 12 born alive, 4 only survived for any length of time.

"Dr. Paterson of Glasgow, and Mr. Heane of Gloucester, succeeded by this means.

"Mr. Corry and Dr. Lee tried it but failed.

"Although the medicine appears successful as regards the induction of labor and the consequences to the mother, yet the proportion of children lost is greater than by the other methods; and this must be a serious objection to its use, when the pelvis will admit the passage of a viable child."

"An interval, varying from twenty-four to ninety-six hours, generally elapses after the operation, before uterine action commences, which it does sometimes by shivering and feverishness. 'Great disturbance in the nervous system,' says Dr. Gooch, 'is produced by it; severe rigors, rapid pulse, and delirium, are the occasional consequences; but these symptoms, proceeding from nervous irritation, do not continue long enough to produce any serious consequences.' In many cases these symptoms are altogether absent. The patient will require the same management as after ordinary labor. It will be advisable to have a nurse in readiness, to supply the infant with its natural nourishment, until the mother shall have milk for it."

---

Manual of the Diseases of the Skin. From the French of MM. Cazenave and Schedel; with Notes and Additions. By *Thomas Burgess*, M.D. Renshaw, 1842.

This is an extremely useful little work, and one which we can safely recommend both to students and medical practitioners. It is founded on the experience of M. Biett, well known during his life as a high authority on cutaneous diseases. The classification adopted is that of Willan, being based on the elementary characters of the disease; certain modifications, however, are, and in most instances with propriety, introduced.

The first, or introductory chapter, affords some very useful hints on the general characters and management of their diseases; the remarks on *diagnosis* are especially deserving of notice.

The chief point is to determine the elementary lesion; we have then merely to compare the disease with the few which possess the same elementary character.

If the elementary lesion is unaltered, we have only to determine the order to which it belongs, and this is usually not difficult; we have then to determine the species, and in this we are aided by the form, seat, &c. of the eruption.

"For example, a patient has, on the inner side of the arm, between the fingers, &c. a number of *small collections of serum*, distinct, accuminated, transparent at the point, and accompanied by itching, &c. On carefully examining, we find that the elevations contain no pus, that they are not solid and resisting, that they are not papular eminences covered by a scale, nor an injection of the skin which disappears under pressure; the disease is therefore *vesicular*. We have then to find out to what species of vesicular affection it belongs. It is neither *miliaria* nor *varicella*, which are accompanied by constitutional symptoms; it is not *herpes*, for in herpes the eminences are collected together in groups; it must therefore be either eczema or scabies; but it is not eczema, for the vesicles of eczema are flattened, while here they are accuminated; *ergo*, it is scabies."

In many cases, however, the elementary character has become lost, and the secondary form presents itself. Thus the fluid of a vesicle may disappear, and leave a small incrustation, a pustule may be converted into a scab, and the latter give way to an ulcer; it is necessary, therefore, that we should be acquainted with these secondary forms, and be able to refer them to their primary states. Incrustations may succeed vesicles, scabs to pustules, and ulcers follow rupia, &c.

"In cases like the foregoing, we must first ascertain the nature of the secondary lesion, then determine its corresponding primary element, and finally pursue the course just pointed out. For example, a patient comes to us with a disease of the skin, characterised by thick, rough, yellow scabs, which cover a large portion of the extremities, especially the legs, and when they fall off expose superficial excoriations; the latter discharge a purulent secrection, which dries up, and forms fresh scabs, these being the most characteristic features of the disease. Now it is easy enough to tell at once that this is a pustular affection, but not so easy to determine its species. The disease is evidently neither *variola* nor *vaccinia;* the pustules of *ecthyma* are large, isolated, and frequently covered by black, tenacious scabs, which end in ulceration; it is neither *acne* nor *mentagra,* the pustules, of which rarely ever give rise to scabs. The only affections, then, that remain are *impetigo* and *porrigo,* and we have merely to compare the character of these two species in order to decide. It is unnecessary to enumerate here the signs by which we know that the disease is not porrigo; it is therefore impetigo, and as the scabs are scattered irregularly over the limb, it is *impetigo sparsa.*"

It is true, that we may not be able always at once to discover the nature of the disease in this way, but still we shall be greatly aided in our diagnosis; at the same time we must neglect nothing which can assist us, as the seat, form, colour, of the eruption, its progress, &c. signs which at once strike the experienced eye, and enable us to dispense with details.

As a specimen of the manner in which the various diseases are treated, we shall transcribe the article upon Ecthyma.

### ECTHYMA.

"SYN.—*Phlyzacia; Agria; Scabies fera; Furunculi atonici.*

"Ecthyma is a disease of the skin, characterised by large round phlyzaceous pustules, almost always distinct, and seated upon a hard inflamed base. These pustules are succeeded by thick, dark-coloured scabs, which leave slight superficial cicatrices behind them, or more frequently, red stains which disappear after a certain time. This eruption may appear on every part of the body, more especially on the neck, the shoulders, the buttocks, the extremities, and the chest. The pustules are seldom developed on the face or on the scalp. Although they are generally distinct from each other, they may, however, spread over a large surface, even over the whole body, but they are usually confined to some particular region.

"*Causes.*—Ecthyma is frequently produced by distinctly apparent causes: it is also sometimes developed spontaneously. In the first instance, it is often the result of irritating applications to the skin: thus, for instance, the characteristic pustules of ecthyma are frequently produced by friction with tartar emetic ointment. The pustules are usually set close together, the epidermis is always elevated for a considerable extent, by a sero-purulent fluid, and this elevation is in general umbilicated. They continue for several days, and are then succeeded by scabs, which begin to form in the centre; the accompanying inflammation is sometimes pretty severe, but it does not occasion any inconvenience, inasmuch as it is often desirable to establish this condition as a curative measure. It must not, however, be allowed to become intense.

"Idiopathic ecthyma is often the result of handling pulverulent and metallic substances; hence it is so frequently seen in grocers and masons. Ecthyma is also developed spontaneously, and in general appears to be symptomatic of some peculiar condition of the economy. It attacks all ages, and appears in every season, but it most frequently appears during the spring and summer in young persons and in adults. Women are sometimes affected with it during pregnancy. It appears to result in the majority of cases from great exertion, fatigue, bad food, want of cleanliness, and intense mental emotions. It is likewise developed in the advanced stages of certain chronic affections of the skin, as lichen, prurigo, and especially scabies; or during the convalescence of some of the acute diseases, as scarlatina, measles, and variola. Finally, chronic inflammation of some of the internal organs may have considerable influence on the production of ecthyma, and in some rare cases an eruption of ecthymatous pustules has appeared during the crisis of gastro-enteritis. Ecthyma may be altogether partial, and confined to one particular spot, when its duration varies from one to two weeks; or it may be general, appearing on every part of the body at the same time, usually by successive eruptions, and continue for weeks and even months.

"*Symptoms.*—When the disease is partial, the eruption appears at once; but it more commonly shews itself in successive crops. It usually commences with the evolution of red, inflamed, circumscribed spots, which attain a considerable size in the course of a few days. Their apices contain pus, whilst their bases are hard, circumscribed, and of a deep red colour. The fluid dries up in two or three days; and pretty thick scabs are formed, leaving dark red stains behind when they fall off. The pustules are in general distinct; they sometimes form irregular groups, and vary in size from that of a pea to that of a shilling, and beyond. The eruption is occasionally accompanied with very severe pain. In some instances suppuration takes place rapidly; in others slowly, not for several days. Sometimes the pus forms in small quantity, and occupies the apex of the pustule alone, the base of which is broad, hard, and inflamed. The epidermis is often raised considerably, so as to form a bulla. The purulent fluid is frequently confined beneath by a thin circular layer of transparent serous fluid. This appearance presents, especially when the pustules are formed on the hands and feet. Some of the pustules terminate by resolution, and slight whitish incrustations appear successively on the surface: but generally they are succeeded by thick, adherent scabs, which, on falling off, leave a deep red patch, and in some rare instances a cicatrix When the eruption is successively developed for a considerable period, the red patches become very numerous and confounded together, giving a peculiar appearance to the diseased surface, which is only to be seen in ecthyma. Sometimes these pustules succeed deep ulcerations, particularly those of the lower extremities, which follow scarlatina and small-pox. They are then greatly inflamed round the base, the scabs are thick, and the ulcerated surface is in general dull, sanious, bloody, painful, and always unhealthy looking.

"Ecthyma frequently occurs in weak, ill-fed, cachectic children, especially during the convalescence of gastro-enteritis, when accompanied with distended abdomen. The size of the pustules is generally very irregular. A small pimple may often be seen close by a large pustule. They are of a circular form, and their colour is more or less red, according as the child is feeble and debilitated. The large pustules frequently suppurate, and, after a lengthened period, terminate in a small cicatrix; but often, after threatening suppuration, they gradually diminish, and terminate by desquamation.

"In old irritable persons, much addicted to drink, a variety of ecthyma is often observed, the *ecthyma cachecticum* of Willan, having much resemblance to rupia. It generally forms on the limbs, but every part of the body is subject to it. The skin is inflamed and more swollen than in the common forms of the disease. It assumes a deep red colour, and in about six or eight days the cuticle is raised over the pustule, is blackish, and infiltrated with blood. It soon bursts, and forms a thick dark scab, raised at the centre; the edges are hard, callous, and more or less inflamed. The scabs are very adherent, and do not become detached for several weeks—sometimes for months. If they fall accidentally, an unhealthy ulceration ensues, and the scab is with difficulty removed. Sometimes febrile symptoms precede or accompany the eruption, but they generally disappear with the disease. Sometimes engorgement of the lymphatic ganglions accompanies this affection, which it will be necessary to reduce by bleeding, &c. Suppuration and desiccation are the usual terminations to ecthyma. Resolution and ulceration are much more rare.

"*Diagnosis.*—The pustules of ecthyma are generally easily recognised by their size and their inflamed base. These characters are sufficient to prevent them from being confounded with those of acne, impetigo, sycosis, and porrigo. However, when the pustules of acne and sycosis are accompanied with a hard red base, as they often are, they might be mistaken for the *phlyzaceous* pustules of ecthyma, if the induration more than the inflamed base of the former, and other peculiarities, which are always to be detected, did not obviate this error. The umbilicated pustules of variola, and the multilocular pustules of vaccinia, together with their contagious nature, will prevent their being confounded with ecthyma.

"It is more difficult to distinguish the eruption of ecthyma from that of syphilis, especially as the latter sometimes presents the same physical characters as the former. In these cases the copper-coloured areola, the history of the case, and the accompanying symptoms, form the basis of our diagnosis. Ecthyma cannot be confounded with scabies, if we recollect that the one is a vesicular and the other a pustular disease; and, if a few pustules should appear amongst the vesicles, the respective characters of scabies and ecthyma will enable them to be distinguished at a glance.

"Ecthyma may be distinguished from furunculi, by bearing in mind, that in the former, the inflammation proceeds from without inwards, whilst in furunculi it commences in the subcutaneous cellular tissue, which becomes mortified to a certain extent. It then proceeds outwards, and forms an opening, by which the dead tissue is expelled. Finally, rupia resembles ecthyma so much, that these two affections often appear to be merely varieties of the same disease. Ecthyma lucidum is much more difficult to distinguish from rupia than the simpler varieties of that disease.

"*Prognosis.*—Ecthyma is not a dangerous affection. The prognosis varies according to the extent of the disease, the age and condition of the patient, and the nature of the accompanying lesions.

"*Treatment.*—When the eruption is mild, partial, and follows a regular course, it merely requires diluents, simple or emollient baths, and attention to diet. If it assumes a severe form, and is accompanied with much inflammation, bleeding, or the application of leeches, may be resorted to with advantage. When the disease is of long standing, and the constitution of the patient is deteriorated, hygienic measures should form the principal part of the treatment. The patient should take moderate exercise, and nourishing food, together with simple or slightly-stimulating baths, as the alkaline or salt-water bath. Mild laxatives are very beneficial. Spirituous liquors, and excesses of all kinds, should be particularly avoided. Tonics, as quinine, iron, &c., are sometimes required. Emollient applications ought to be employed when the ulcers are inflamed, and difficult to heal. It is sometimes necessary, on the other hand, to excite the surface with nitrate of silver, or some stimulating lotions. Muriatic acid, diluted with water, is very efficacious in altering the condition of the parts, which under this treatment assume a more healthy aspect, and soon cicatrize."

---

A PRACTICAL TREATISE ON PULMONARY CONSUMPTION, ITS PATHOLOGY, DIAGNOSIS, AND TREATMENT, &c. By *Francis Cooke*, M.D. London, Churchill. 1842.

Dr. Cook states in his Preface that the principal object which he has in view in adding one to the enormous quantity of books which have appeared on this subject of late years, is, to inquire into the causes of the failure of the ordinary course of treatment adopted, and to ascertain "why this disease has not participated, equally with others, in the improvements which have taken place in medical practice of late years." As chemistry has advanced new remedies have been discovered adapted to diseases formerly considered as almost beyond the reach of the physician's art. In like manner, our author is inclined to imagine, consumption may prove to be not altogether the intractable disease which it is usually thought to be.

Dr. Cooke's views upon the subject appear to be these. Recovery from consumption may take place either in the first, or in the second stage of the disease:—In the first, by absorption, by which the tubercle is removed while yet in a crude state; in the second, by the softening and expectoration of the tuberculous matter, and the formation of a cicatrix or of a semi-cartilaginous cavity in the substance of the lung. How then are we to assist Nature in procuring one or other of these results?

The indications of cure in the first stage, are:—

1. To improve digestion, &c. and thus prevent the formation of fresh tubercle.
2. To promote the absorption of tubercle already deposited.
3. To allay pain, cough, &c., arrest hectic and emaciation, and remove any inflammatory complication or hæmorrhage which may exist.

1. With regard to the first point, Dr. Cooke thinks that "the blood of the consumptive being deficient in vitality, their food should be of such a quality, and taken in such a quantity, as to compensate for this deficiency. This end is most readily obtained by the lighter and more digestible kinds of animal nutriment: while the drink should be moderately stimulating, so as to afford some assistance to the stomach during the progress of digestion." With regard to change of climate, this is of benefit in this stage, in those cases where there is a great susceptibility to atmospheric variation. Great care ought to be taken in choosing the place to which the invalid is to be sent.

2. To promote absorption of tubercle;—though we know of no medicine which can effect this directly, yet there are some agents which may fulfil this end by indirectly stimulating the pulmonary absorbents. These are mercury, iodine and antimony. Of these Dr. Cooke seems to prefer iodine, applied to the lungs in the state of vapour. For this purpose, a teaspoonful of the iodine solution (gr. ij. iv. ad ℥ij.), may be added to half a pint of water at the temperature of 180, and the patient directed to inhale the vapour for five minutes thrice a day. If the cough is troublesome, a little conium or hyoscyamus may be added. "The good effects of these measures are often apparent at the end of a fortnight; but I may here inculcate the necessity of a steady perseverance in the plan of treatment recommended."

3. To increase the tone of the system, and diminish irritability and hectic fever, Dr. Cooke most strongly recommends iron. Hectic fever, he says, is usually assignable to debility or exhaustion, occurring in an irritable habit, or in one rendered so by disease. Salines, &c. must not therefore be given merely because the affection is termed fever; "the nature, not the name of the disease must regulate its treatment."

This treatment consists in giving iron, many formulæ for the administration of which, are given. This medicine, Dr. Cooke is inclined to think, not merely acts by increasing the hæmatozine or red particles of the blood, and so augmenting the vital energy, but also, not improbably, may possess a "specific or peculiar power of counteracting the tuberculous diathesis; being, as it were, the antidote to the morbid element or poison."

To cause an increased determination of blood to the surface, relieving irritation and removing internal irritation, it is useful to rub the chest with some stimulating liniment.

To stop hæmoptysis, Dr. Cooke is decidedly adverse to the employment of bleeding; light diet, cold drinks, digitalis, antimony, or the acetate of lead, should be employed. "By these measures, the heart's action and the pulmonary circulation may be controlled as effectually as if the patient had been bled to insensibility; while the hæmorrhagic re-action, which always follows depletion, is avoided; and the vital power, of which there is always sufficient need during convalescence, is spared."

"The preceding is the treatment of phthisis incipiena, which occasionally proves fatal; the processes of respiration and sanguification being impeded by the quantity of tubercles which fill the lungs. Violent attacks of dyspnœa occur, and the patient sinks before ulceration or any great emaciation has taken place. But more frequently the tubercles which occupy the superior portions of the lungs ulcerate, and are discharged by expectoration, forming the second stage of consumption, or phthisis confirmata."

*Treatment of the Second Stage of Phthisis.*—The indications of cure here, are, 1st, To promote the cicatrization of the tuberculous cavities; and 2nd, To relieve cough, diarrhœa, hectic, &c.

As it is impossible that the cavities in the lungs can cicatrize until they are emptied of their contents, the first point is to promote expectoration. Nature endeavours to effect this by the cough, it is improper therefore to interfere with this unnecessarily. Inhalations of the vapour of water containing a small quantity of hops, or of sulphuric æther, occasionally act better than any expectorant. When the cough is irritating, with but little expectoration, watery vapour diffused through the room, with perhaps the addition of some narcotic or emollient herbs, is often attended with relief. The air of all the rooms moreover, ought to be kept at an uniform temperature. Dr. Cooke very properly reprobates the practice of sending patients in the last stage of phthisis, when no hope of recovery can exist, to die in a foreign land, separated from their friends and relations, and deprived of all the comforts of home.

In the Appendix, Dr. Cooke gives a very concise and useful tabular view of the physical signs of phthisis in its two stages; and another, exhibiting the annual mean temperature, height of the barometer, and weather, of the most noted places of resort for consumptive patients.

Having thus completed our analysis of the work, we have only to state that it is a very neatly written little book, and contains several useful hints as to the treatment of this most distressing malady, though Dr. Cooke undoubtedly carries his objections to the "lowering" system a little too far.

---

### TRANSACTIONS OF THE MEDICAL AND PHYSICAL SOCIETY OF BOMBAY FOR THE YEAR 1841. No. IV. Bombay, 1841.

### ARTICLE II.

REPORT ON THE DISEASES WHICH HAVE OCCURRED IN THE H. C.'S STEAM FLOTILLA, ON THE RIVERS OF MESOPOTAMIA, FROM THE 1ST JANUARY TO THE 31ST DECEMBER, 1839, WITH A SHORT SKETCH OF THE CLIMATE AND TOPOGRAPHY. By JOHN C. FLOYD, Esq., Surgeon in Charge.

*Presented by the Medical Board, April,* 1841.

THIS is an excellent number; for, besides other articles of interest and importance, it contains five very able ones upon Topography, Climate, and Medical Statistics—the subjects most eagerly sought after in Europe, because by far the most important and interesting on the spot, in Asia.

The necessity, in respect to public health, of considering something more than is to be gathered from tables of the winds, and of the thermometric and barometeric ranges and averages, is now beginning every where to be felt. We now begin to view man in relation to all these causes, whether moral or physical, that exercise an influence on his habits, or on his health, including a comprehensive detail of his social and political relations: in short, we now consider that we must know every thing that relates to the man, so far as our apprehension and the state of science admit, ere we can be satisfied that we are really acquainted with his diseases, or their causes.

If, as we say, such is the state of feeling in this great question, in the region wherein we are now writing, with all its advantages, natural and acquired, and wherein man has made his own climate the pre-eminently good one that it now is, how much more does the subject increase in importance, owing to the concentrated nature of the causes of disease, so as to demand an increased and a careful consideration from our brethren in the East—the East, where man, owing to moral and social causes, has hardly yet made a step towards ameliorating his physical condition, without which, in the first instance, he must continue for ages yet to come, and despite of European institution and example, the same civil and religious slave he has been for ages past; and the Hindoos can count their fifty centuries of this double abjectness.

These preliminary observations are offered in commendation and encouragement of the efforts now, we hope, about to be made on a great and extended scale, by our brethren, to elucidate *the external causes of disease* in the East Indies. We can assure them, as their brother officer, Mr. Martin, has already done—that "such objects are in reality of more value than volumes of cases or details of routine practice; their careful investigation will confer permanent benefits on the public service, and, sooner or later, derive honor to themselves, difficult if not impossible to be obtained in any other way."

But we must now direct attention to Mr. Floyd's excellent sketch of the medical topography of the banks of the Euphrates and surrounding country, comprising the southern division of Turkish Arabia.

"Before entering on the above report, I have thought it right to preface it with a short sketch of the topography and climate of the country: for, the latter are so intimately connected with the production of disease, that without a knowledge of them, it would be as difficult to understand the various diseases, extending over such a tract of country, as it would be to localize any particular one.

The country, to which this report refers, extends from 30° to 34° North latitude, and from 40° to 45° East longitude. It includes all the country between the Persian Gulph and Bagdad on the North, the Lauristan Hills on the East, and the desert to the West of the Euphrates; and it has borne successively the names of Shinar, Babylonia, Chaldæa, Mesopotamia, and Irack Arabi. It forms at present the southerly division of Turkish Arabia, and comprises the greater part of the Pachalic of Bagdad.

Its physical aspect is that of an immense plain, composed of a deep stratum of black alluvial soil, watered by the Euphrates and Tigris, and intersected with numerous canals. These two great rivers, rising in the mountains of Armenia, pursue a course of several hundred miles, and then emerge from their rocky channel, about one hundred miles above Bagdad on the Tigris, and fifty above Hilla on the Euphrates. From thence, after a serpentine course of four hundred and fifty miles, during which they often approach within thirty miles of each other, they unite at Korna, (said to be one of the ancient "Apameæ," built by Seleneus Nicator, now an Arab village,) forty miles above Bussora, forming one of the noblest rivers of the East, which, under the name of the Shut-il-Arab, runs one hundred and thirty miles further, and disembogues itself into the Persian Gulf: the current averages two knots in the low, and five in the high season.

These rivers, in their extensive course through a flat country, are constantly changing their character, breaking down their banks, and forming islands, and these sharing the same fate every succeeding year.

The quantity of debris brought down every year, cannot be calculated with certainty, as it must vary with the rise and fall of the stream; but, it is immensely great, there can be no doubt, from the rapid formation of islands and banks, and also from the immense alluvial banks, which are gradually extending themselves into the Persian Gulf.

*Surrounding Country.*—This is exceedingly bare, vegetation scanty, except on the banks of the rivers, where the pastoral Arabs feed their flocks, and in some places have settled down to cultivate the soil.

The soil from Bagdad to the Persian Gulf is entirely alluvial, and of a depth varying from twenty to thirty feet, we then come to a blue and white clay of a very adhesive nature, which contains a quantity of silica and alumina. This is manufactured into vases and water-vessels, and from its porosity, allowing of slight evaporation from the surface, the water contained in them is always deliciously cool.

No rocks any where show themselves, but from the character of the hills North of Bagdad and Hilla, their dip and strike into the Mesopotamia plain, I have reason to believe that they consist of a micaceous slate, gypsum, and calcareous sand-stone, the second of these abounding. Muriate of soda is an extensive product of this desert, and particularly near Bussora, where it is observed in the form of beautiful chrystals, at the edge of every little pond, and it is exported in large quantities from the place. The immense ruins, composed of sun and kiln-burnt bricks, which are scattered over this country, attest the greatness of its former population; the earth at these ruins, as that of Seleucia below Bagdad, when washed and evaporated, yields a considerable quantity of saltpetre.

*Canals.*—The number and extent of these extraordinary works, which once rendered this country so productive, we cannot do more than glance at. We are told that, "in consequence of the periodical rise of the Euphrates, artificial canals were dug by the ancient inhabitants, for the double purpose of protecting the contiguous plains from inundation, and at the same time of preserving the superfluous waters for the irrigation of the soil;"—that it had this effect, the present sterility of the upper portion of this country, and the inundation of the lower, fully demonstrate. Between Bagdad and Babylon, a distance of forty-five miles South, we meet with four very celebrated canals, one of these called the Nahar Malacha, or Royal canal, which Julian sailed his fleet through. The magnitude of this may be judged from the fact of the Honorable Company's steamer Euphrates having sailed through one called the Succaloweia, which runs from river to river, but it is now shut up to prevent any further inundation. Another canal, called the Naharawan, runs on the East side of Bagdad for a distance of three hundred miles; its bed is nearly dry. Midway between Bagdad and Bussora, at Kool-il-Amarah, is a canal called the Heye, which runs to the Euphrates, for a distance of ninety miles; it is navigable.

The only other canal which I shall mention is the famous Palla Copus, navigated by Alexander the Great, and supposed to be the ancient bed of the Euphrates. It commences below Hilla, and a branch of it was repaired for a short distance by the Nabob of Oudi as far as Mesid Ali, a place of pilgrimage. This canal being stopped up, the Euphrates has overflowed its banks, and formed an extensive, and deep lake, in the western desert.

*Vegetation,*—as might be expected, is abundant on the banks, particularly in the vicinity of Bussora, Korna, Bagdad, and Hilla, where forests of splendid date-trees confer on the inhabitants immense wealth; along with these the glycyrrhiza, the tamarisk, and the urunda abound, and in some places the two latter form immense jungles, in which the lion and the leopard are occasionally seen. The gardens at Bussora and Bagdad produce almost all the varieties of fruit, peculiar to a temperate climate, and even some of the tropical plants flourish here, as the mango, the plantain, and herma: all the European culinary vegetables are likewise yielded in abundance. As rain does not fall in sufficient quantities for agricultural purposes, all irrigation is artificial, and carried on by means of water-wheels worked by bullocks. The different sort of corn, sesamum orientale, (an oil plant,) are cultivated in great abundance, also cotton, opium, indigo, rice, and tobacco.

The Delta enclosed between the Euphrates and Tigris, has, not unaptly, been compared to that of Egypt; for in the time of Herodotus, the ground yielded three hundred and sixty times more than it received, and whatever may be the approach to truth, when oriental historians fix upon it as the site of the "Garden of Eden," certain it is, that its capabilities are as evident as ever, though the caperbush, the colocynth (of the Pharmacopœia), some species of mimosa, the camelthorn, the salix Babylonica, and a few saliferous plants, are all that now relieve the eye, in a country which once yielded produce equal to a third part of Asia.

*The Inundations.*—The banks of these rivers are exceedingly low, and thereby easily overflowed, at the time of the annual rise; these take place in the Spring and Summer months, from the melting of the snow in the mountains of Armenia; they form an important feature in the medical topography of this country, as they completely change the nature of the climate where they prevail. The rivers often fall and rise during the Winter months, but the grand rise takes place in the Tigris in April, and in the Euphrates in May, when the former rises about eighteen feet, and the latter fourteen feet, and the current which before was only one and a half, is increased to five knots. Notwithstanding this, the rivers keep their height, the banks then give way, and a more or less extensive inundation covers the country, depopulating villages, and laying waste the gardens and fields; such, at least, was the case at Bagdad in 1839, when from the above cause half the town was under water, but such are temporary and soon retire, as the rivers again descend in August and September.

From Bagdad and Hilla northwards the country is not so liable to these inundations, as the banks are higher. About thirty miles above the junction of the rivers at Korna the desert is always one sheet of water. Carry a line from this point across the Euphrates, and from thence over the desert to Bussora and Tobier, it is one vast marsh for a distance of seventy miles. This inundation took place about eleven years ago, shortly before the last plague. A line of banks was formerly kept up along the Euphrates to keep the water out, but these have been completely swept away, and the government of the country are neither able nor willing to repair them, which would recover a fine tract of country, secure Bussora from ruin and the inhabitants from disease. Indeed it is not improbable that the Euphrates will find its way into its ancient channel, which emptied itself near the modern town of Grane in the Persian Gulf.

*Climate.*—The climate of this country must vary exceedingly, as we descend from the snow-clad hills where the rivers have their source; but, of that portion, between the Persian Gulf and Bagdad, included in this report, there is little difference unless where local causes operate. The seasons may be divided into Spring, hot, and cold; the former commences about the end of January, and continues till the end of April. The thermometer ranges from 50° to 70° daily, though sometimes it rises much above this; North and South winds blow alternately for five or six days at a time, and at their change sometimes heavy showers of rain descend and renew the verdure of the desert: all agricultural operations are now carried on. May is the first of the very hot months; in it the corn ripens, and is gathered in before the end of June, when the thermometer averages 105° daily, and everything appears scorched from the sun. The heat of July and August is intolerable—the Arabs call it "saum," or the date harvest; the thermometer is 115° in the shade, and the inhabitants take shelter in their surdaubs or cellars. Hot winds occasionally prevail; the nights however are cool; the atmosphere free from moisture, except near the marshes; and refreshing sleep in the open air compensates, in some degree, for the enervation produced by the heat of the day. In September the heat gradually declines. The cold weather commences in October, when there is a variation of 20° to 25° daily, and from this to the end of January it is not unusual to see everything frozen on the decks. The prevailing winds are the north-west and south-east, called by the natives the Shumaul and Shirgee; they blow chiefly in the Winter and Spring. Tornadoes or whirlwinds are not uncommon in the hot weather; they approach giving but little warning, and carrying an immense cloud of dust which involves everything in obscurity. It was in such a one that His Majesty's steamer "Tigris" was lost on the Euphrates in 1835.

The following Table taken from the register of the thermometer kept in the cabin of the Euphrates, which is rendered very cool by wind-sails, will elucidate the above."

TABLE, shewing the Maximum, Minimum, and Mean Temperature, as registered from the Thermometer at Bagdad, for the Year 1840.

| | Maximum. | Minimum. | Mean. | Prevailing Winds. |
|---|---|---|---|---|
| January ...... .. | 64 | 25 | 50 | N.W. and S.E., with rain. |
| February ........ | 68 | 36 | 57 | N.W. and S.E., with little rain. |
| March .......... | 75 | 36 | 66 | Strong N.W. |
| April ........... | 83 | 48 | 74 | Ditto. |
| May ........... | 95 | 65 | 77 | Towards the end of the month hot wind. |
| June............ | 101 | 72 | 87 | N.W. breezes. |
| July ............ | 105 | 78 | 98 | Calm clear weather, thermometer 105° underneath the awnings, and 135° in the sun. |
| August.......... | 108 | 79 | 97 | Hot winds. |
| September ...... | 100 | 65 | 85 | N.W., temperature of river 80°. |
| October......... | 97 | 58 | 67 | S.W., with squalls. |
| November ....... | 76 | 51 | 63 | Rains with squalls. |
| December ....... | 65 | 33 | 45 | In the fore part of this month N.W. with rain, in the latter frosts." |

This very excellent description is followed by a more detailed reference to the particular stations of the steamers; and this last, by an account of the prevailing diseases. These are just what might, *a priori*, be expected to arise in such a country as we have seen described, acted on by a powerful sun. The observations of Mr. Floyd on the several diseases, their prevention and cure, are highly judicious and practical; but we cannot now afford time or space to refer to them.

We hope in another number to recur to the Bombay "Transactions," and to notice the other excellent articles contained in it. Meanwhile, we trust to be favoured with many succeeding volumes, comprising articles such as we have here briefly noticed.

